# Self‐Reported Everyday Psychosocial Stressors Are Associated With Greater Impairments in Endothelial Function in Young Adults With Major Depressive Disorder

**DOI:** 10.1161/JAHA.118.010825

**Published:** 2019-02-12

**Authors:** Jody L. Greaney, Rachel E. Koffer, Erika F. H. Saunders, David M. Almeida, Lacy M. Alexander

**Affiliations:** ^1^ Noll Laboratory Department of Kinesiology The Pennsylvania State University State College PA; ^2^ Department of Human Development and Family Studies The Pennsylvania State University State College PA; ^3^ Department of Psychiatry Penn State College of Medicine Hershey PA

**Keywords:** cardiovascular disease risk factors, depression, nitric oxide, vascular endothelial function, Endothelium/Vascular Type/Nitric Oxide, Vascular Biology, Pathophysiology

## Abstract

**Background:**

Despite the epidemiological associations between psychological stress, depression, and increased cardiovascular disease risk, no studies have examined the relation between naturally occurring psychosocial stressors and directly measured microvascular function in adults with major depressive disorder (MDD). We tested the hypothesis that young adults with MDD exposed to everyday psychosocial stressors would exhibit more severe impairments in endothelium‐dependent dilation (EDD) compared with: (1) healthy nondepressed adults (HCs); and (2) adults with MDD without acute psychosocial stress exposure.

**Methods and Results:**

Twenty HCs (22±1 years) and 23 otherwise healthy adults with MDD (20±0.3 years) participated in the study. Participants completed a psychosocial experiences survey to document their exposure to any of 6 stressors over the preceding 24 hours (eg, arguments, work stressors). Red cell flux (laser Doppler flowmetry) was measured during graded intradermal microdialysis perfusion of acetylcholine (10^−10^ to 10^−1^mol/L). EDD was expressed as a percentage of maximum vascular conductance (flux/mm Hg). Multiple linear regression was used to determine the associations between stress, EDD, and MDD. Adults with MDD reported a greater number and severity of psychosocial stressors compared with HCs (all *P*<0.05). EDD was blunted in adults with MDD (HCs: 91±2 versus MDD: 74±3%; *P*<0.001). Exposure to any stressor was related to more severe impairments in EDD in patients with MDD (no stressor: 81±3 versus 1+ stressors: 69±5%; *P*=0.04) but not in HCs (*P*=0.48).

**Conclusions:**

These data indicate that exposure to everyday psychosocial stressors is associated with greater impairments in endothelial function in patients with MDD, suggesting a potential mechanistic link between daily stress and depression in increased cardiovascular risk.

**Clinical Perspective**

**What Is New?**

- The novel finding of this study is that acute exposure (within 1 day) to any naturally occurring everyday psychosocial stressor is associated with greater impairments in microvascular endothelial function in treatment‐naive young, otherwise healthy, adults with major depressive disorder.

**What Are the Clinical Implications?**

- These data suggest that: (1) microvascular endothelial dysfunction may be a potential mechanism linking daily psychosocial stress and depression in contributing to increased cardiovascular disease risk; and (2) strategies to promote stress resistance and resilience in adults with major depressive disorder may mitigate or prevent stress‐induced impairments in microvascular function.

## Introduction

Despite recent declines in prevalence, cardiovascular disease (CVD) remains the leading cause of death and disability worldwide.^1^ Traditional risk factors (eg, high blood pressure, smoking, and dyslipidemia) alone do not fully explain CVD risk, and, in Western society, there is increasing awareness that social, environmental, and psychological factors are mediators of, and may even directly cause, CVD.^2^ A large body of evidence indicates that long‐term exposure to psychosocial stress contributes to the development of CVD.^3^, ^4^ The largest study supporting this connection demonstrated that psychosocial stress is independently associated with increased risk of acute myocardial infarction, and, further, the increase in cardiovascular risk posed by the presence of psychosocial stress is similar in magnitude to that of traditional CVD risk factors.^5^ Despite this compelling epidemiological evidence, the specific mechanisms by which psychosocial stress‐induced pathophysiological processes impart increased CVD risk remain incompletely understood.

Exposure to acute stressors includes activation of the hypothalamus‐pituitary‐adrenal axis and the autonomic nervous system.^6^, ^7^ Activation of these systems elicits a myriad of physiological responses aimed at protecting the body from a perceived threat, including increases in blood pressure and heart rate, energy mobilization, and immune activation.^6^, ^7^ Although the acute stress response has been well described, less is known regarding how exposure to a psychosocial stressor translates to pathological alterations that contribute to the development and progression of CVD. One prevailing concept is that the integrated response to acute stress induces transient endothelial dysfunction^8^, ^9^, ^10^, ^11^—thought to be the first functional manifestation of atherosclerosis and a primary causative event in the development of overt CVD.^12^ Collectively, chronic and repetitive exposure to psychosocial stress, or a maladaptive response to repeated stressors (ie, exaggerated stress “reactivity”), via its deleterious effects on the vascular endothelium, may accelerate the atherosclerotic process and precipitate the development of CVD.^13^

Chronic or repeated exposure to psychosocial stress also plays a critical role in the pathogenesis of many mood disorders, including major depressive disorder (MDD),^8^, ^14^, ^15^, ^16^ a pathology that is now considered a nontraditional CVD risk factor.^17^, ^18^ The Intern Health Study prospectively followed a cohort of medical student trainees from the low stress period before internship to the very high stress period during the internship year.^19^ The dramatic increase in stress during medical internship was associated with a substantial increase in the rate of depression from <5% before internship to ≈25%.^19^ Coincident with the increase in depressive symptoms was a significant reduction in endothelial function,^19^ substantiating the links between exposure to stress, the development of depressive symptoms, and detrimental alterations in vascular function. However, medical internship represents a relatively atypical and severe occupational stress, thereby limiting the applicability of these findings. Thus, the aim of the present investigation was to examine the modulatory influence of common everyday psychosocial stressors (eg, arguments and home stressors) on direct measures of microvascular endothelial reactivity in depression, thereby providing insight critical to our understanding of stress vulnerability. We hypothesized that treatment‐naive, otherwise healthy, young adults with MDD exposed to everyday psychosocial stressors would exhibit more severe impairments in microvascular endothelium‐dependent dilation (EDD) compared with: (1) healthy nondepressed adults (HCs), and (2) adults with MDD without acute psychosocial stress exposure.

## Methods

### Participants

The institutional review board at The Pennsylvania State University and the Food and Drug Administration (IND 125,994) approved all experimental procedures. Verbal and written consent were obtained voluntarily from all participants before participation and in accordance with the guidelines set forth by the Declaration of Helsinki. The data that support the findings of this study are available from the corresponding author upon reasonable request.

Forty‐three adults participated (Table 1). All participants underwent a complete medical screening, including physical examination, diagnostic psychiatric interview, resting blood pressure and heart rate measurements, and 12‐hour fasting blood chemistry (Quest Diagnostics). Participants were free of cardiovascular, metabolic, renal, and neurological disease; were nonobese (body mass index <30 kg/m); did not use tobacco products; were recreationally active; and were not taking any prescription medications with primary or secondary cardiovascular effects. A urine pregnancy test confirmed the absence of pregnancy. Five women (2 HCs and 3 with MDD) used hormonal contraception. The timing of the experimental visit was not controlled for menstrual cycle phase because, with this experimental protocol, there does not appear to be an effect of menstrual cycle phase on vascular endothelial function.^20^, ^21^ Moreover, patients with MDD were tested within ≈1 week of the screening visit to facilitate expedient follow‐up with a mental healthcare provider.

**Table 1:** Patient Characteristics

	HC	MDD
No. (men/women)	20 (10/10)	23 (9/14)
Age, y	22±1	20±0.3^a^
Height, cm	173±2	171±3
Mass, kg	71±3	67±3
BMI, kg/m^2^	23.8±0.8	22.8±0.8
Heart rate, bpm	66±1	67±2
Screening systolic BP, mm Hg	118±3	114±3
Screening diastolic BP, mm Hg	73±1	72±2
Experimental systolic BP, mm Hg	120±2	118±2
Experimental diastolic BP, mm Hg	76±2	73±2
Total physical activity (MET‐min/wk)	7752±2543	7443±2268
Blood biochemistry		
Hemoglobin, g/dL	14.1±0.4	13.4±0.3
Hematocrit, %	42.4±1.1	40.5±0.9
Glucose, mg/dL	90±2	94±3
HbA_1c_, %	5.3±0.04	5.0±0.2
Fasting total cholesterol, mg/dL	162.4±8.4	148.8±6.0
Fasting HDL, mg/dL	61.1±3.9	55.2±3.6
Fasting LDL, mg/dL	74.8±6.0	72.7±4.6
Fasting triglycerides, mg/dL	83.1±6.1	91.3±11.2
Depression symptom severity		
PHQ‐9	1±0.4	12±1^a^
Psychosocial stress variables (range)		
Experience any stressor, No. (%)	16 (80)	12 (55)
No. of stressors, 0 to 6	0.8±0.2	2.0±0.3^a^
Stressor severity, 0 to 3	1.5±0.2	2.0±0.1^a^
Stressor‐related negative emotions, 0 to 4	0.2±0.05	0.4±0.04^a^
Rumination about stressor, 0 to 3	0.8±0.2	1.4±0.2^a^
Negative affect, 0 to 4	0.2±0.1	1.2±0.1^a^

Values are expressed as mean±standard error. BMI indicates body mass index; BP, blood pressure; bpm, beats per minute; HbA_1c_, glycated hemoglobin; HDL, high‐density lipoprotein; LDL, low‐density lipoprotein; MDD, major depressive disorder; MET, metabolic equivalent; PHQ‐9, Patient Health Questionnaire.

a *P*<0.05 vs healthy nondepressed adults (HCs).

### Diagnostic Assessment

All participants underwent the structured Mini‐International Neuropsychiatric Interview,^22^ and a diagnosis of MDD was confirmed by a psychiatrist according to the fifth revision of the *Diagnostic and Statistical Manual of Mental Disorders* (*DSM‐5*) criteria.^23^ Patients with comorbid current psychiatric disorders (eg, psychosis, schizophrenia, bipolar disorder, panic disorder, and obsessive compulsive disorder), active suicidal ideation (moderate or high suicidality), or using psychoactive or psychopharmacological drugs within 1 year were excluded from participation. Five adults with MDD also presented with generalized anxiety disorder. Given the high comorbidity of MDD with generalized anxiety disorder,^24^ these adults were not excluded from study participation. Depressive symptom severity was evaluated using the Patient Health Questionnaire‐9, which provides a valid and sensitive index of symptomology based on the diagnostic criteria for *DSM‐5* depressive disorders.^25^

### Assessment of Psychosocial Stressors

During the experimental visit, participants completed a modified version of the Daily Inventory of Stressful Events (DISE) to document exposure to any of 6 naturally occurring everyday psychosocial stressors (ie, daily hassles) over the preceding day^26^: arguments/disagreements, avoidance of arguments/disagreements, work/school stressors, home stressors, experience that happened to a close friend/relative that was stressful for the participant, or any other stressful event. Obtaining information about daily psychosocial stressors over this short‐term interval alleviates concerns regarding ecological validity and retrospective memory distortions that can occur over longer time frames.^26^ Moreover, daily psychosocial stress assessed using a telephone version of this interview has been linked to changes in depressive symptoms over 10 years^27^ and with diurnal cortisol,^28^ a biomarker for stressor reactivity.

Self‐reports to each type of stressor were used to compute 2 stressor exposure indicators: (1) *number of stressors* is the total number of stressors reported, and (2) *experiencing any stressor* is a binary indicator of whether the individual reported having at least 1 stressor during the previous week. For each stressor, participants also rated the severity of the stress, the amount of control they felt over the stressor, and the amount of time spent thinking about the stressor (all on a scale of 0=not at all to 3=very), as well as whether (yes=1, no=0) the stressor contributed to any of 4 negative emotions: angry, sad, nervous, shameful. The mean of these indicators across all reported stressors was used to compute each individual's average stressor severity, rumination over stressors, and stressor‐related emotions. The modified DISE also documented the duration (on a scale of 0=none of the time to 4=all of the time) of 14 affective states in order to calculate average negative affect.^26^

### Laboratory Assessment of Microvascular Endothelial Reactivity

One intradermal microdialysis probe (CMA Linear 30 probe, 6 kDa; Harvard Apparatus) was inserted into the dermal layer of the ventral forearm using sterile technique for the local delivery of pharmacological agents, as previously described in detail.^29^, ^30^, ^31^ Pharmacological agents were mixed immediately before use, dissolved in lactated Ringer solution, filtered using sterile syringe microfilters (Acrodisc, Pall), and wrapped in foil to prevent photodegradation. Pharmacological agents were perfused through the microdialysis probes at 2 μL/min (Bee Hive controller and Baby Bee microinfusion pumps, BASi). Red blood cell flux, an index of cutaneous blood flow, was continuously measured directly over each microdialysis site with an integrated laser Doppler flowmeter probe placed in a local heating unit (VP12 and VHP2, Moor Instruments) set to thermoneutrality (33°C), unless otherwise noted. Automated brachial blood pressure (Cardiocap, GE Healthcare) was measured every 5 minutes throughout the protocol.

The experimental protocol began after an initial ≈60 to 90 minutes hyperemia‐resolution period. After baseline measurements, increasing concentrations of acetylcholine (10^−10^ to 10^−1^ mol/L, United States Pharmacopeia), an endothelium‐dependent agonist, were sequentially perfused for 5 minutes each to ensure a plateau. Following the ACh dose‐response protocol, 28 mmol/L sodium nitroprusside (United States Pharmacopeia) was perfused and local temperature was increased to 43°C to elicit maximal dilation.^29^, ^30^, ^31^, ^32^

### Data and Statistical Analysis

Red blood cell flux was recorded at 40 Hz (PowerLab and LabChart, ADInstruments). Vascular conductance was calculated as laser Doppler flux (perfusion units) divided by mean arterial pressure, normalized as a percentage of the maximum (%_max_), and averaged during 5 minutes of baseline and during the last minute of each acetylcholine dose. Vascular conductance was analyzed using 2‐way (group×dose) and 3‐way (group×sex×dose) mixed model repeated‐measures ANOVA (SAS version 9.4), with post hoc Bonferroni corrections applied for specific planned comparisons when appropriate.

Multiple linear regression (or ANOVA for the 1 categorical variable) was used to determine the associations between self‐reported psychosocial stress variables, depression status, and EDD at the highest concentration of acetylcholine. Specifically, for each psychosocial stress variable, a regression was fit as follows:

$$\text{Endothelium}-\text{dependent}\;{\text{dilation}}_{i}=\beta_{0}+\beta_{1}\text{Psychosocial}\;{\text{Stress}}_{i}+\beta_{2}\text{Depression}\;{\text{Group}}_{i}+\beta_{3}\text{Psychosocial}\;{\text{Stress}}_{i}*\text{Depression}\;{\text{Group}}_{i}+\varepsilon_{i}$$

where MDD was the reference group for depression status, so β_0_ is the EDD for a depressed participant with the sample's mean value of the psychosocial stress variable, β_1_ is the unique association between the psychosocial stress variable and EDD for a depressed participant, β_2_ is the unique association between healthy control status and EDD, β_3_ is the extent to which the psychosocial stress variable modulates the association between depression status and EDD, and ε_*i*_ is the unexplained residual that is assumed to be independent and normally distributed. All continuous variables were mean centered, and all missing data were treated as missing at random. A follow‐up ANOVA was performed to test whether sex modulates the association between any significant stressor variables, depression status, and endothelial function. Significance was set at α<0.05. Data are presented as mean±standard error of the mean.

## Results

Participant groups were well matched for anthropometric characteristics, resting cardiovascular parameters, habitual physical activity, and blood biochemistry (Table 1; all *P*>0.05). Adults with MDD were significantly younger than HCs (*P*=0.01). Adults with MDD were experiencing a major depressive episode of mild to moderate severity (Table 1; *P*<0.01).

### Daily Psychosocial Stress is Greater in MDD

Psychosocial stress variables are also displayed in Table 1. There were no group differences in experiencing at least 1 psychosocial stressor in the preceding 24 hours (*P*=0.08). Adults with MDD reported a greater number of stressors, stressor severity, stressor‐related negative emotions, and rumination about stressors compared with HCs (all *P*<0.05), supporting the established link between psychosocial stress and MDD.^27^ As expected, adults with MDD reported a higher negative affect compared with HCs (*P*<0.001).

### Microvascular EDD is Blunted in MDD

**Figure 1:**
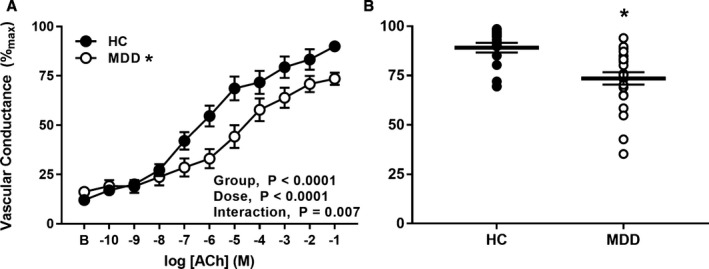
Vascular conductance in response to graded perfusion of acetylcholine (**A**) and during perfusion of the highest concentration of acetylcholine (**B**) in healthy nondepressed adults (HCs, n=20; filled symbols) and in adults with major depressive disorder (MDD, n=23; open symbols). Endothelium‐dependent dilation was blunted in patients with MDD. Data are mean±standard error. *P<0.05 vs HC.

Baseline (HCs: 0.23±0.03 versus MDD: 0.27±0.04 PU/mm Hg; *P*=0.36) and maximal vascular conductance (HCs: 2.0±0.17 versus MDD: 1.85±0.11 PU/mm Hg; *P*=0.49) were not different between groups. Vasodilation in response to perfusion of the endothelium‐dependent agonist acetylcholine is presented in Figure 1. As expected, EDD was blunted in MDD, assessed as both the full range of responsiveness to graded perfusion of acetylcholine (*P*<0.0001; Figure 1A) and maximal acetylcholine‐induced dilation (HCs: 91±2 versus MDD: 74±3%_max_; *P*<0.01 [Figure 1B]), reflective of pronounced microvascular endothelial dysfunction. There were no sex differences in EDD in either group (HC men: 86±4 and HC women: 95±2; MDD men: 75±6 and MDD women: 73±4%_max_ [*P*=0.15]).

### Psychosocial Stress is Related to Greater Impairments in EDD in Patients With MDD

**Figure 2:**
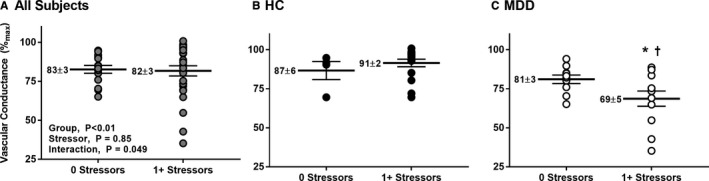
Vascular conductance during perfusion of the highest concentration of acetylcholine in patients who experienced any psychosocial stressor in the preceding week (1+ stressors) and in those who did not (0 stressors) for all patients (gray symbols; **A**), healthy nondpressed adults (HCs, n=20; filled symbols; **B**), and adults with major depressive disorder (MDD, n=23; filled symbols; **C**). There was a significant interaction between experiencing any psychosocial stressor and depression status in predicting endothelial function (*P*=0.049). Exposure to a psychosocial stressor was related to more severe impairments in endothelium‐dependent dilation in MDD (*P*=0.04) but not in HCs (*P*=0.48). Data are mean±standard error. *P<0.05 vs HCs; ^†^ *P*<0.05 vs 0 stressors.

There was no unique effect of experiencing any psychosocial stressor on EDD (*P*=0.85, Figure 2A); however, there was a significant interaction between experiencing any psychosocial stressor and depression status in predicting endothelial function (β=−17.28, *P*=0.049). Experiencing any psychosocial stressor in the preceding day did not affect EDD in HCs (*P*=0.48, Figure 2B). In contrast, in patients with MDD, experiencing any stressor was related to more severe impairments in acetylcholine‐induced dilation (*P*=0.04, Figure 2C). EDD was not blunted in adults with MDD who did not experience a psychosocial stressor compared with HCs (*P*=0.43). Sex did not modulate the relation between experiencing any stressor and depression status in predicting endothelial function (*P*=0.96). Although additional aspects of psychosocial stress were correlated with EDD (number of stressors *r*=−0.24, stressor severity *r*=−0.27; stress‐related negative emotions *r*=−0.50, rumination *r*=−0.24, negative affect *r*=−0.39; all *P*<0.05), none were modulated by depression (Table 2, all *P*_β3_>0.05).

**Table 2:** Relationship Between Psychosocial Stress and Depression in Mediating Endothelial Function

Psychosocial Stress Variable	Intercept β_0_ (SE)	Stressor β_1_ (SE)	Group β_2_ (SE)
No. of stressors	74.1 (2.9)^a^	0.3 (1.8)	16.6 (4.5)^a^
Stressor severity	73.8 (3.4)^a^	−2.6 (4.1)	13.7 (5.9)^a^
Stressor‐related negative emotions	75.2 (3.2)^a^	−31.4 (15.2)^a^	10.1 (5.7)
Rumination about stressor	73.7 (3.4)^a^	−1.8 (3.2)	14.0 (5.8)^a^
Negative affect	75.6 (5.2)^a^	−1.1 (3.7)	15.2 (5.3)^a^
Positive affect	75.1 (3.7)^a^	1.3 (3.8)	14.4 (6.6)^a^

SE indicates standard error.

a *P*<0.05.

## Discussion

As expected given the established link between psychosocial stress and depression,^27^ in the current study, young adults with MDD reported experiencing a greater number and severity of psychosocial stressors than their HC counterparts. Further, EDD was markedly impaired in adults with MDD, consistent with the concept that depression is characterized by pronounced endothelial dysfunction.^33^, ^34^, ^35^, ^36^, ^37^, ^38^ The novel finding of the current study is that acute exposure (within 1 day) to any naturally occurring everyday psychosocial stressor is associated with greater impairments in microvascular endothelial function in treatment‐naive young otherwise healthy adults with MDD. Taken together, these findings suggest a potential mechanistic link between naturally occurring daily stress and depression in contributing to increased CVD risk.

Psychosocial stress is an independent risk factor for CVD,^5^ yet there is still a relatively limited understanding of the mechanistic mediators of this association. Accumulating evidence indicates that psychological stress adversely impacts vascular endothelial function.^10^, ^39^, ^40^, ^41^ For example, in healthy adults free of cardiovascular risk factors, a psychological stress task chosen to approximate the type of stress likely to be encountered frequently during normal daily activities (ie, after given 2 minutes to prepare, participants orally presented a 3‐minute defense against a false shoplifting accusation in front of an audience) acutely impaired brachial artery flow‐mediated dilation.^10^ This acute impairment appears transient, as endothelial function returned to baseline at 2 hours following the psychological stress task.^10^ Given the notion that endothelial dysfunction causally contributes to the development of atherosclerosis,^12^ the acute stress‐induced deleterious effects on the endothelium may represent a physiological link between psychological stress and CVD risk.^3^, ^4^ Chronic and repetitive exposure to psychological stress also plays a critical role in the pathogenesis of many mood disorders, including MDD.^8^, ^14^, ^15^, ^16^ Similar to psychosocial stress, depression has also been linked to the excessive and premature development of CVD, independent of traditional risk factors,^17^, ^18^ and a growing body of evidence supports endothelial dysfunction as a potential mediator of depression‐CVD comorbidity.^33^, ^34^, ^35^, ^36^, ^37^ However, despite this evidence for an independent association between stress and, separately, depression, and endothelial dysfunction, surprisingly few studies have attempted to discern the interrelation between daily psychological stress, depressive symptomology, and endothelial function.

The majority of studies that have examined psychological stress‐ or depression‐related alterations in vascular function have utilized reactive hyperemia‐induced increases in shear stress in a conduit artery to assess EDD.^10^, ^39^, ^40^, ^41^ Although reductions in brachial artery flow‐mediated dilation appear to be predictive of cardiovascular events in asymptomatic individuals,^42^ deficits in vascular function in the *micro*circulation are among the earliest indicators of CVD pathogenesis. As such, microvascular dysfunction is a better predictor of long‐term outcomes and adverse cardiovascular events than large‐vessel disease.^43^, ^44^ In the present study, we assessed endothelial function in the cutaneous microcirculation, an easily accessible vascular bed and a validated model for generalized microvascular dysfunction in multiple pathologies.^44^, ^45^, ^46^, ^47^, ^48^ Moreover, this approach allows for the targeted pharmaco‐dissection of signaling pathways mediating microvascular endothelial function and dysfunction.

Consistent with the available literature reporting large artery dysfunction in adults with depression,^33^, ^34^, ^35^, ^36^, ^37^ this study documented attenuated EDD in response to direct pharmacological stimulation in the microvasculature of adults with MDD. In addition, and as expected,^27^ adults with MDD reported experiencing a greater number and severity of psychosocial stressors in the day preceding the assessment of endothelial function compared with HCs. Moreover, exposure to at least 1 psychosocial stressor was related to more severe impairments in microvascular endothelial function in adults with MDD. Although all other indices of psychosocial stress were greater in patients with MDD, neither the number of stressors, stressor severity, rumination over stressors, nor stressor‐related negative emotions were related to endothelial dysfunction in patients with MDD. Interestingly, it appears that exposure to psychosocial stress may be necessary in order for depression‐related decrements in endothelial function to become overt, because although qualitatively reduced, statistical differences in vasodilation in response to acetylcholine were not observed between HCs and adults with MDD who did not experience a stressor. When considered collectively, we interpret these data as providing support for the concept that the exposure to a common, naturally occurring everyday stressor, but not necessarily the perceived severity of that stressor, predicts more severe endothelial dysfunction in patients with MDD. That is, simply experiencing a psychosocial stressor appears to have short‐term detrimental consequences for microvascular endothelial function that are only evident in young adults with MDD. Further, the relative absence of psychosocial stress may be vasoprotective for adults experiencing depression.

These findings are consistent with those reporting that psychological stress, in the form of an acute laboratory mental stress task, modulates the relation between depressive symptomology and impairments in flow‐mediated dilation.^49^ Although acute psychological stressors applied in the laboratory setting (eg, mental arithmetic and public speaking) evoke a clear cardiovascular response and provide important information regarding mental stress‐induced alterations in physiological function, there are inherent limitations with this approach. Laboratory assessments of psychosocial stress often lack ecological validity using artificial stimuli that rarely occur in day‐to‐day life. The exposure to these laboratory stressors is relatively short‐term (eg, minutes) and often presented the same in all of the respondents.^50^ One of the few studies to examine the interrelation between “real‐world” psychological stress, depression, and vascular function was the Intern Health Study.^8^ This prospective study attempted to establish the temporal relation between change in depressive symptomology and vascular outcomes. Using medical internship as a model of psychological stress, the authors reported that an increase in stress exposure was linked to the development of mild depressive symptomology, which, in turn, was inversely correlated with changes in reactive hyperemia index obtained in the finger,^8^ such that participants with the greatest increase in depressive symptoms demonstrated the greatest impairment in endothelial function. Although medical internship stress may be considered a short‐term psychological stressor that occurs in the context of daily living, it is relatively atypical and severe. Thus, to extend these findings to a more generalizable index of psychosocial stress, we utilized a modified version of the DISE^26^ to document exposure to any of 6 naturally occurring everyday psychosocial stressors during the day preceding the experimental measurement of microvascular reactivity. This approach to assessing psychosocial stress, which allows for the measurement of the content and severity of everyday stressors, has been validated on a national sample of adults and correlated with negative mood and physical health symptoms.^26^ Importantly, we are the first to: (1) utilize this approach to assess psychosocial stress in otherwise healthy young adults with MDD, and (2) relate self‐reported exposure to daily psychosocial stress with microvascular endothelial dysfunction.

Interestingly, the experience of a psychosocial stressor does not appear to detrimentally influence microvascular EDD in young HCs. The lack of an effect of psychosocial stress on endothelial function is perhaps surprising in light of evidence reporting that acute laboratory‐applied psychological stress impairs EDD.^10^, ^39^, ^40^, ^41^ It is important to note that stress‐induced endothelial dysfunction is not a universal finding,^51^, ^52^ and, even in some studies reporting a negative association,^10^ not all patients demonstrated decreased EDD following psychological stress. Although the mechanisms contributing to this variability are incompletely understood, it is increasingly evident that the psychological health of the research participants may be important in predicting the vascular response to stress.^53^ The data in the current study provide support for this idea, as a negative association between psychosocial stress exposure and endothelial function was only evident in adults with clinically apparent alterations in their psychological functioning.

The downstream mechanisms converting psychosocial stress into cellular dysfunction and vascular disease are still largely unknown. Although likely complex and multifactorial, one plausible candidate is via the activation of the redox‐sensitive transcription factor nuclear factor‐κB (NF‐κB),^54^ which is a critical downstream regulator of the inflammatory and redox state of vascular endothelial cells and whose pathological activation directly contributes to atherogenesis.^55^ Both rodent and human studies have described stress‐induced increases in NF‐κB activation.^54^, ^56^ Moreover, it appears that an adrenergic signaling pathway contributes to the rapid increase in activation of NF‐κB following exposure to psychosocial stress.^54^ Interestingly, increased psychological stress–induced NF‐κB pathway activity has been noted in adults with MDD.^57^ Because NF‐κB activation is also directly linked to vascular endothelial dysfunction in adults with increased CVD risk,^58^ this inflammatory signaling pathway warrants future targeted investigation as a potential mechanism mediating the link between psychosocial stress, depression, and microvascular dysfunction. However, it is important to note that the extent to which the immediate response elicited by a psychosocial stressor is sufficient to mediate the link between stress and CVD remains unclear. Although our data provide evidence that acute (within 1 day) exposure to psychosocial stress adversely influences endothelial function in MDD, it is likely that repeated and chronic exposure to stressful life events, coupled with a maladaptive or pathophysiological stress response, is requisite to more fully explain heightened CVD risk in depression.

The timing of the experimental visits for women was not controlled for menstrual cycle phase, which is a limitation. To our knowledge, no studies have examined the influence of female sex hormones on endothelial reactivity to perfusion of acetylcholine to the cutaneous microvasculature via intradermal microdialysis in healthy young women or young women with MDD. The studies that have assessed cutaneous EDD in relation to menstrual cycle phase have utilized direct iontophoresis of acetylcholine, and the findings from these limited studies are equivocal.^20^, ^59^, ^60^ As such, the influence of female sex hormones on microvascular endothelial reactivity to acetylcholine remains unclear. Even less is known regarding the relation between menstrual cycle phase and microvascular endothelial function in young women with MDD. Interestingly, rodent models of depression demonstrate that endothelial dysfunction is apparent throughout the estrous cycle, despite a varied hormone profile.^61^ The lack of sex differences in the present study, despite the fact that women were not studied at the same point of their menstrual cycle, has significant implications. These results suggest that MDD‐induced vascular impairments in women may not be critically dependent on the specific time within the menstrual cycle but may instead be relatively insensitive to the fluctuations in hormonal profiles. Nevertheless, targeted investigation of the influence of menstrual cycle phase and female sex hormones on microvascular function in young women with depression is clearly necessary.

### Conclusions and Perspectives

The concepts of stress resistance and resilience have been an increasing focus of attention in stress physiology research, particularly in relation to clinical mood disorders. This is especially relevant given the sheer heterogeneity of both the disease process of mental illness itself as well as in an individual's responsiveness to stress.^62^ Stress resistance is commonly thought of as the duration and/or intensity of stressor exposure that is requisite for crossing from an adaptive to a maladaptive stress response.^63^ Stress resilience, on the other hand, refers to the facilitation of a recovery in function once the stress response has become maladaptive.^63^ In this light, a maladaptive stress response can result not only in the development of clinical mood disorders themselves but also in the initiation and progression of—and an inability to recover from—stress‐induced pathophysiological alterations in cardiovascular function. Our data suggest not only that microvascular endothelial dysfunction may be a potential mechanism linking daily psychosocial stress and depression in contributing to increased CVD risk but also that strategies to promote stress resistance and resilience in adults with depression may mitigate or prevent stress‐induced impairments in microvascular function. In other words, strategies to increase the exposure to psychosocial stress that an adult with MDD can endure before experiencing negative vascular consequences, as well as to facilitate the recovery of function following stressor exposure, may be necessary in addition to traditional pharmacological approaches to manage depressive symptoms. Indeed, cognitive behavioral therapy and habitual aerobic exercise both yield stress‐buffering outcomes thought to occur via neural adaptations critical for evoking increases in stress resistance and resilience.^63^ Whether targeting the mechanisms by which these adaptations occur also informs our understanding of the neurobiology of depression itself is an exciting venue for future research.

## Sources of Funding

This work was supported by National Institutes of Health (NIH) awards HL093238 (Alexander), HL133414 (Greaney), T32 AG049676 (Koffer), the National Center for Advancing Translational Sciences UL1 TR002014, and an American Heart Association award 16SDG30240006 (Greaney). The content is solely the responsibility of the authors and does not necessarily represent the official views of the NIH.

## Disclosures

None.
